# Neonicotinoid pesticides can reduce honeybee colony genetic diversity

**DOI:** 10.1371/journal.pone.0186109

**Published:** 2017-10-23

**Authors:** Nadège Forfert, Aline Troxler, Gina Retschnig, Laurent Gauthier, Lars Straub, Robin F. A. Moritz, Peter Neumann, Geoffrey R. Williams

**Affiliations:** 1 Institute of Biology, Martin-Luther-University Halle-Wittenberg, Halle (Saale), Germany; 2 Institute of Bee Health, Vetsuisse Faculty, University of Bern, Bern, Switzerland; 3 Agroscope, Swiss Bee Research Centre, Bern, Switzerland; 4 Social Insect Research Group, Department of Zoology and Entomology, University of Pretoria, Pretoria, South Africa; 5 Department of Entomology and Plant Pathology, Auburn University, Auburn, Alabama, United States of America; University of North Carolina at Greensboro, UNITED STATES

## Abstract

Neonicotinoid insecticides can cause a variety of adverse sub-lethal effects in bees. In social species such as the honeybee, *Apis mellifera*, queens are essential for reproduction and colony functioning. Therefore, any negative effect of these agricultural chemicals on the mating success of queens may have serious consequences for the fitness of the entire colony. Queens were exposed to the common neonicotinoid pesticides thiamethoxam and clothianidin during their developmental stage. After mating, their spermathecae were dissected to count the number of stored spermatozoa. Furthermore, their worker offspring were genotyped with DNA microsatellites to determine the number of matings and the genotypic composition of the colony. Colonies providing the male mating partners were also inferred. Both neonicotinoid and control queens mated with drones originating from the same drone source colonies, and stored similar number of spermatozoa. However, queens reared in colonies exposed to both neonicotinoids experienced fewer matings. This resulted in a reduction of the genetic diversity in their colonies (i.e. higher intracolonial relatedness). As decreased genetic diversity among worker bees is known to negatively affect colony vitality, neonicotinoids may have a cryptic effect on colony health by reducing the mating frequency of queens.

## Introduction

Pollinating insects provide important ecosystem and economic services by foraging on wild plants and agricultural crops [1]. Recent reports about the decline of wild pollinators, and high annual mortality of managed honeybees, have raised concerns for food security and the maintenance of biodiversity [1]. Habitat loss and fragmentation, climate change, pests and pathogens, alien species, and agrochemicals have been listed as potential causes of these losses [2,3].

Neonicotinoids are neurotoxic insecticides that are ubiquitously employed in agriculture for pest control. The widespread use of such neurotoxic insecticides results in residual accumulation of low concentrations in the environment [4,5]. Acting as agonists on nicotinic acetylcholine receptors (nAChRs) of the insect central nervous system [6], their presence leads to hyperactivity of the neuronal system [7,8]. This can result in both lethal and sublethal effects in bees [9–13]. The majority of studies that have investigated the potential effects of neonicotinoids on honeybees focused on the worker caste (primarily non-reproductive females); they demonstrated adverse effects on cognition (e.g. learning, memory, sense perception) [9,14–16], behaviour (e.g. foraging, homing, mobility) [17–20] and physiology (e.g. muscle activity, larval development) [8,21]. However, disparities between lab and field results, possibly due to experimental methods (e.g. exposure routes, treatment concentrations), remain contentious [22]. Although workers are essential for colony functioning, the queen, which typically monopolises reproduction, is the single most important individual in a colony, and essential for its persistence, particularly when emergency queen rearing is not possible [23]. Even though eusocial insects show super-organismic resilience against stressors [24], any effects on queens may have profound consequences for the entire colony.

Given the various effects of neonicotinoids on honeybees, it is possible that queens may be particularly susceptible to neonicotinoids during the demands of mating [25]. Honeybee queens are polyandrous, typically mating on average with 12 male drone partners [26,27]. Mating occurs on the wing within three weeks post-emergence at Drone Congregation Areas (= DCAs) [28,29], which are typically located up to 5 km away from the virgin queen’s colony [30]. Immediately after each mating event, the oviducts of the queen can be filled with up to 200 million spermatozoa, but only ~7 million will migrate posteriorly with the assistance of muscular contractions to a special storage organ called the spermatheca [31,32]; residual spermatozoa are excreted [33]. The release of spermatozoa from the spermatheca for egg fertilization is rigorously controlled by the sperm pump [31,34]; upon depletion of spermatozoa the queen will be superseded and killed by the colony [23].

Extreme polyandry of the honeybee queen results in lowering average relatedness among workers within a colony, which translates into greater genetic diversity. The ability of a queen to mate with multiple drones is paramount to her own the fitness, as well as the fitness of the colony. Extreme polyandry may benefit the colony for various reasons [35], including the availability of sufficient spermatozoa for colony maintenance [36,37], improved colony efficiency [38–40], improved adaptation and response to environmental changes [41–45], and reduced disease intensity [38,46].

Poor queen quality has been frequently observed by beekeepers, and is considered to be a major driver of overwintering colony mortality [47]. Recently, Williams et al. [48] reported that queens exposed to 4 ppb of thiamethoxam and 1 ppb of clothianidin exhibit reproductive anatomical (larger number of ovaries) and physiological abnormalities (lower quantity and quality of stored spermatozoa), as well as reduced success (survival and oviposition); no effect on behaviour (flight duration and number) was observed. Similarly, queens were more often superseded in honeybee colonies exposed to thiamethoxam and clothianidin [49].

Given that factors affecting queen mating can affect colony productivity [38–40], and because reduced queen health, possibly because of poor mating, is frequently cited as a major cause for colony death [50], we studied the effects of field-realistic concentrations of the combination of two neonicotinoids, thiamethoxam and clothianidin, on queen mating and genetic diversity among worker offspring. Both pesticides are widely used globally to control a range of insects [51], but their application to pollinator attractive crops is currently subject to a partial moratorium by the European Commission [52]. We compared mating frequencies of neonicotinoid-exposed and control queens using microsatellite DNA genotyping. We report for the first time that neonicotinoids can affect honeybee intracolonial genetic diversity by affecting mating frequency.

## Materials and methods

### Ethics statement

Our study did not involve endangered or protected species.

### Queen rearing

We used the same six *A*. *m*. *carnica* colonies reported by Williams et al. [48]. Three to five experimental queens were obtained from each experimental colony using standard apiculture queen rearing techniques [53]. For this, the original queens were removed from their respective experimental colonies 27 days post initial treatment exposure to create queenless cell-builders. One day later, one-day old larvae were grafted to artificial queen cells on queen cell bar frames in each colony, and placed back into their respective colonies to develop. Two days prior to emergence, queen cells were moved to the laboratory and maintained in an incubator at 34.5°C and 60% relative humidity until emergence [54]. Each reared virgin queen was immediately transferred to one of 24 mini hives (APIDEA) containing 300 g food (APIFONDA®) and 100 g workers (~750 individuals) originating from the virgin queen’s original mother colony. They were confined for three days at 12°C in darkness to promote colony formation, and then placed outdoors to allow for natural open-air mating with drones from the surrounding environment for four weeks.

### Pesticide treatment

Colonies were fitted with hive entrance pollen traps to limit external pollen foraging, and fed daily 100 g honey/pollen (3:1) patties *ad libitum* for 36 days to ensure that young nurse workers exposed to the experimental treatments during their entire development period were available for queen rearing. Three control colonies received patties free of neonicotinoids and three treatment colonies received patties spiked with 4 ppb thiamethoxam and 1 ppb clothianidin (both Sigma-Aldrich; concentration verified by UHPLC-MS/MS at the French National Centre for Scientific Research to be 4.16 and 0.96 ppb, respectively). This is within the concentration ranges found in pollen of treated crops [55,56]. Hence, nurse bees were exposed to treatments during their entire development before engaging in queen rearing. Our neonicotinoid treatment included both thiamethoxam and clothiandin because the latter is a major metabolite of the former [57,58]. Therefore, both can co-occur in the pollen of thiamethoxam-treated crops. Furthermore, this treatment exposure scenario allows for comparison with previously published work [48,49,59].

### Spermatozoa quantification

Queens were collected four weeks post initial oviposition. Spermathecae were removed and placed in Kiev buffer [60]. The number of spermatozoa stored in each spermatheca (Sperm Count) was estimated using a hemocytometer and light microscopy [61].

### Newly emerged bee DNA amplification and genetic analysis

We genotyped 20–24 worker offspring per mated queen that emerged seven weeks post oviposition initiation.

DNA was extracted using a Chelex protocol [62]. Five closely linked microsatellite loci (Table 1) were used to infer parental genotypes [63] using Mendelian inference. Multiplex PCRs were used to amplify 10 ng of DNA in 1 μl DNA dilution buffer (Qiagen), 400 pM of each primer, 1.25x reaction buffer (Sigma), 200 μM of each dNTP, 1U of *Taq*-polymerase and HPLC water to a final volume of 10 μl. The temperature profile for the PCR was as follows: 5 min denaturation at 95°C, 35 cycles of 30 sec each for denaturation (95°C), annealing T_m_ (Table 1) and extension (72°C), followed by a final step of 5 min at 72°C. The amplified products were separated in a MegaBace automated sequencer and fragment sizes were analyzed using the Fragment Profiler software. Alleles were scored as fragment lengths in base pairs.

**Table 1:** Microsatellite markers for honeybee *Apis mellifera* genotyping.

Name	Size (bp)	Dye	T_m_ (°C)	Primer I (5'-. . .-3')	Primer II (5'-. . .-3')	Allelic Diversity (±SEM)
SV240	265	TET	55	CGTGCGCCCTTTTTGTCAC	CGGGACGGTTGATGATGAAG	3.08±0.25
HB004	198	HEX	55	CAAACAAACCGTGTGGATGT	ACTGCGAGGAAAAAGGAAGT	4.08±0.22
HB007	131	HEX	52	TACGACCCATAACACGCAAT	GTTCGTGCCACCTTCTATTC	7.71±0.32
HB015	129	FAM	52	CGGTCGAGAGATGGTTGTAA	GTCATCCACTTTTCCCTTCA	3.00±0.17
HB005	221	TET	52	CGTTTCTCTACCCTCGAACA	ATCTGCCGAAAAGACTCTCA	4.54±0.60

For each primer used to determine queen and drone genotypes from newly emerged offspring, the product size (in bp), the primer dye, the annealing temperature (T_m_ in°C), the pair sequences, and the allelic diversity (number of alleles per colony for 20–24 individuals genotyped ±SEM), are given [64].

### Data analysis

When all workers carried the same allele at one locus, the queen was assumed to be homozygous at that locus. Because males may contribute unequally to future offspring, the number of matings does not reflect the intracolonial genetic diversity. We therefore determined the number of genetically effective matings (= Observed Effective Mating: the genetically effective number of drones if all were equally represented in the queen’s offspring) *m*_*e*_ as follows [42]:

$$m_{e}=\frac{1}{\sum q_{i}^{2}}$$ (1)

where *q*_*i*_ the proportion of offspring sired by the *i*^*th*^ male.

We calculated the Observed Relatedness *r* using [65]:

$$r=0.25+0.5*\sum q_{i}^{2}=0.25+\left(\frac{1}{2m_{e}}\right)$$ (2)

where *m*_*e*_ = Observed Effective Mating and *q*_*i*_ the proportion of offspring sired by the *i*^*th*^ male.

Effective number of matings (*m*_*e*_) and average intracolonial genetic relatedness (*r*) both reflect intracolonial genetic diversity. However, in the highly polyandrous honeybee, *r* does not measure intracolonial genetic diversity well as it quickly approaches the limit of *r* = 0.25 when the number of matings increase.

To correct for non-sampling error, we employed [65]:

$$\sum q_{i}^{2}=\frac{N\sum y_{i}^{2}-1}{N-1}$$ (3)

Where *y*_*i*_ is the observed contribution of each male and *N* is the sample size. By using the estimate calculated from Eq (3), we could calculate the Corrected Effective Mating and the Corrected Relatedness from Eqs (1) and (2), respectively.

Additionally, we determined the Paternity Skew, *S*, of each colony; this reflects the degree of paternity bias among offspring due to post-copulatory sexual selection and sexual conflict [66]. Paternity Skew was calculated as follows [67]:

$$S=\frac{Nt-(\frac{1}{\sum q_{i}^{2}})}{Nt-1}$$ (4)

Where *Nt* is the total number of actual patrilines and *q*_*i*_ the proportion of offspring sired by the *i*^*th*^ male. *Nt* was obtained by adding the number of undetected patrilines to the total number of observed patrilines. The number of undetected patrilines was estimated by using the frequency distribution of the observed patrilines found in the offspring sample, assuming equal distribution of all father drones. Through a fitted Poisson distribution we calculated the frequency for zero, which is the number of undetected patrilines [61].

To determine the non-detection error (*NDE*), which is the probability of obtaining two identical genotypes in two different individuals by chance, we employed [61]:

$$NDE=\sum q_{i}^{2}+\sum r_{i}^{2}+\cdots \sum z_{i}^{2}$$ (5)

Where *q*_*i*_ are the allele frequencies at the first locus, *r*_*i*_ are the allele frequencies at the second locus, and *z*_*i*_ are the allele frequencies at the last locus.

### Statistics

Normality assumptions were tested by using the Shapiro-Wilk’s statistic, while homogeneity of variances was confirmed by using residuals plots. Two-level generalized regression mixed models with random intercepts were fitted using STATA14 [68], wherein individual queens were considered to be independent factors, treatment (neonicotinoid vs. control) as the fixed term, and mated queen source colony as a random effect. For Observed Effective Mating, Corrected Relatedness, Sperm Count and Paternity Skew, the models were fitted using the meglm function. For Corrected Effective Mating and Observed Relatedness, the models fitted the mepoisson function (Table 2). Furthermore, for pollen patty consumption, a three-level generalized regression mixed model with random intercepts was fitted using the non-parametric menbreg function. It included treatment as the fixed term, and colony and time as random effects (Table 2).

**Table 2:** Summary of statistical methods and results.

							95% Confident Interval	
Variable	Treatment	Shapiro-Wilk W	STATA14 Function	P-Value	Type	Regression Coefficient	Lower	Upper
Observed Effective Mating	Control	0.95	meglm	0.0004	linear	-2.53	-3.94	-1.12
	Neonicotinoid							
Observed Relatedness	Control	0.02	mepoisson	0.0004	poisson	0.04	0.02	0.06
	Neonicotinoid							
Corrected Effective Mating	Control	0.05	mepoisson	0.006	poisson	- 2.17	-3.72	-0.62
	Neonicotinoid							
Corrected Relatedness	Control	0.29	meglm	0.003	linear	0.01	0.005	0.025
	Neonicotinoid							
Sperm Count	Control	0.72	meglm	0.151	linear	-0.72	-1.7	0.026
	Neonicotinoid							
Paternity Skew	Control	0.005	meglm	0.628	linear	0.08	-0.52	0.087
	Neonicotinoid							
Patty Consumption	Control	<0.001	menbreg	0.458	binomial	1.02	0.768	1.37
	Neonicotinoid							

Summarized here are the STATA14 functions used to fit two-level models, the outcome variables, types of regression employed, estimated coefficients and 95% CIs, and P-values.

Honeybee queens are expected to fly 1 to 2 km to mate [69], while drones typically locate the closest DCA to their mother colony (~900 m distance) [70]. The genetic pool of drones present in a DCA gives a representation of the local colonies [71]. Therefore, queens that mate with genetically related drones most likely mated in similar DCAs. To assess whether drone producing colonies (i.e. Drone Source Colony) participated similarly to the mating of queens from both treatments, Drone Source Colonies were determined by inferring the original queen genotype of a sampled colony from the father drone genotypes. Since queens are diploid and lay unfertilized eggs that develop into drones, queens have two haplotype copies of the linked microsatellite markers with a highly specific allele sequence. Hence, they produce two types of haplotypes (drones). However, due to the extremely high rate of recombination in the honeybee genome [72], meiotic recombination within the linkage group rearranges the marker sequence so that it is highly specific to the mother queen’s genotype. If the number of drones sampled per colony is sufficient to identify such recombination events, this allows inference of the original queen genotype of a sampled colony and accurate assignment of all her drone offspring [71].

Some drones will only occur as a singleton representing a unique haplotype. In these cases, it is impossible to infer the complete diploid genotype of the original queen. Two singletons can either be the offspring of a single diploid queen or from two different queens. Therefore, we estimated the maximum number of Drone Source Colony by considering singletons originating from a unique colony, and the minimum number of Drone Source Colony by pairing singletons. In this second estimation, singletons were paired either by considering that they mated with the same type of queen (control or neonicotinoid) or not. To estimate the number of Drone Source Colony that remained undetected because of finite sample size (“non-sampling error”) [65], we fitted the empirical mating events of the assigned Drone Source Colony to a Poisson distribution.

The proportion of neonicotinoid and control queens that mated with drones originating from each Drone Source Colony was determined using Fisher’s exact test.

## Results

**Fig 1:**
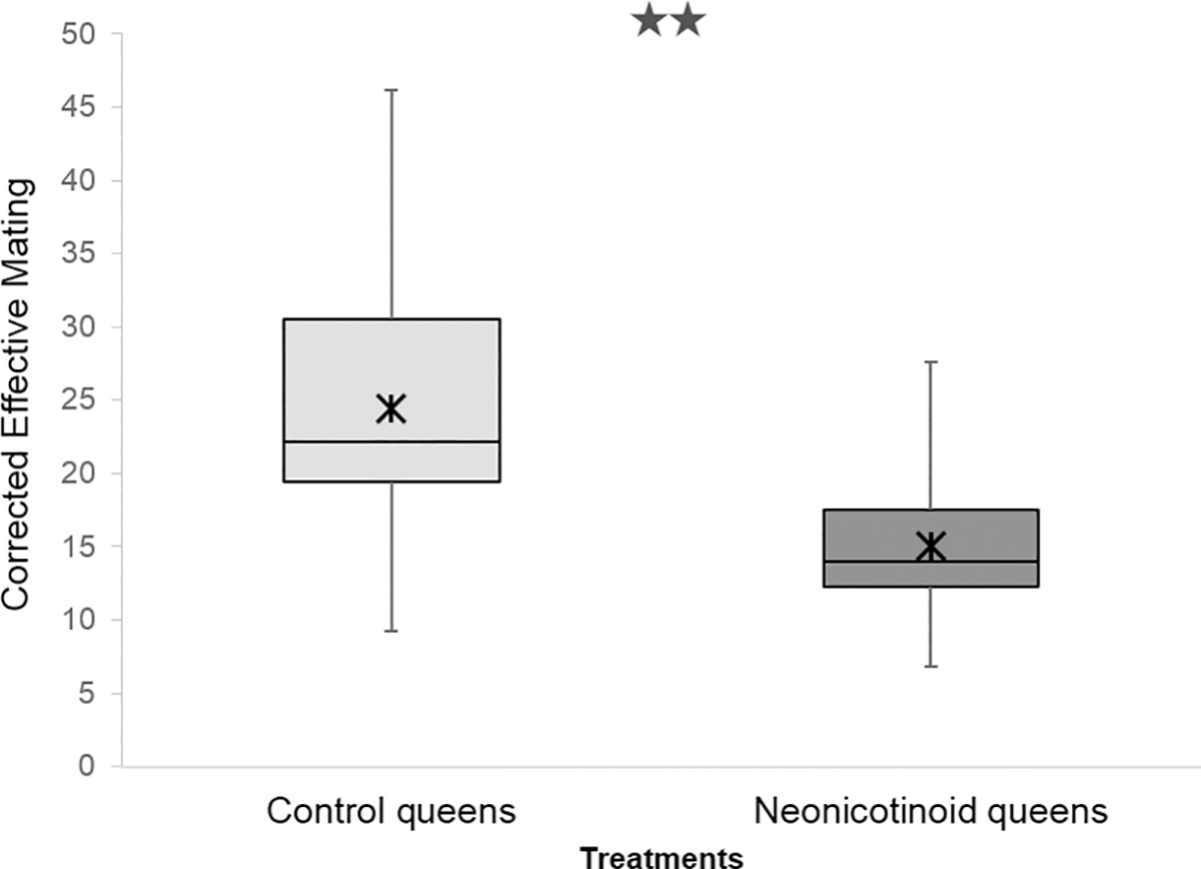
Corrected Effective Mating inferred from offspring DNA genotyping of control and neonicotinoid honeybee queens (*Apis mellifera*). **Boxplot shows inter-quartile range (box), median (black line within interquartile range), means (black asterisk); data range (dashed vertical lines).** Queens exposed to neonicotinoid pesticides during their developmental stage mated with fewer males, resulting in lower Effective Matings than control queens. *P≤0.1, **P≤0.05, ***P≤0.01 (comparison with Controls).

**Fig 2:**
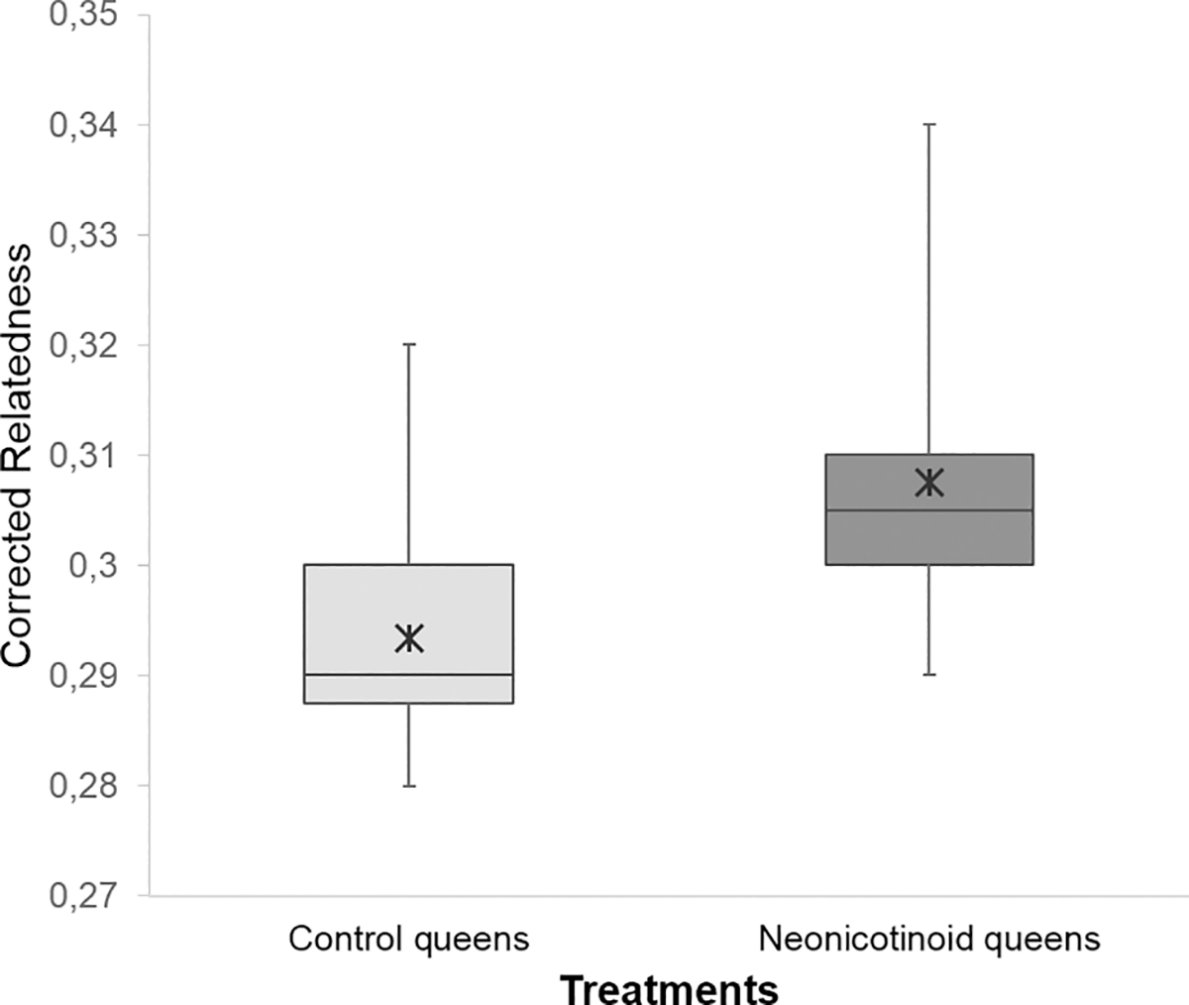
Corrected Relatedness inferred from offspring DNA genotyping of control and neonicotinoid honeybee queens (*Apis mellifera*). **Boxplot shows inter-quartile range (box), median (black line within interquartile range), means (black asterisk); data range (dashed vertical lines).** Queens exposed to neonicotinoid pesticides during their developmental stage mates with fewer males, resulting in higher Corrected Relatedness among worker offspring than control queens. *P≤0.1, **P≤0.05, ***P≤0.01 (comparison with Controls).

**Fig 3:**
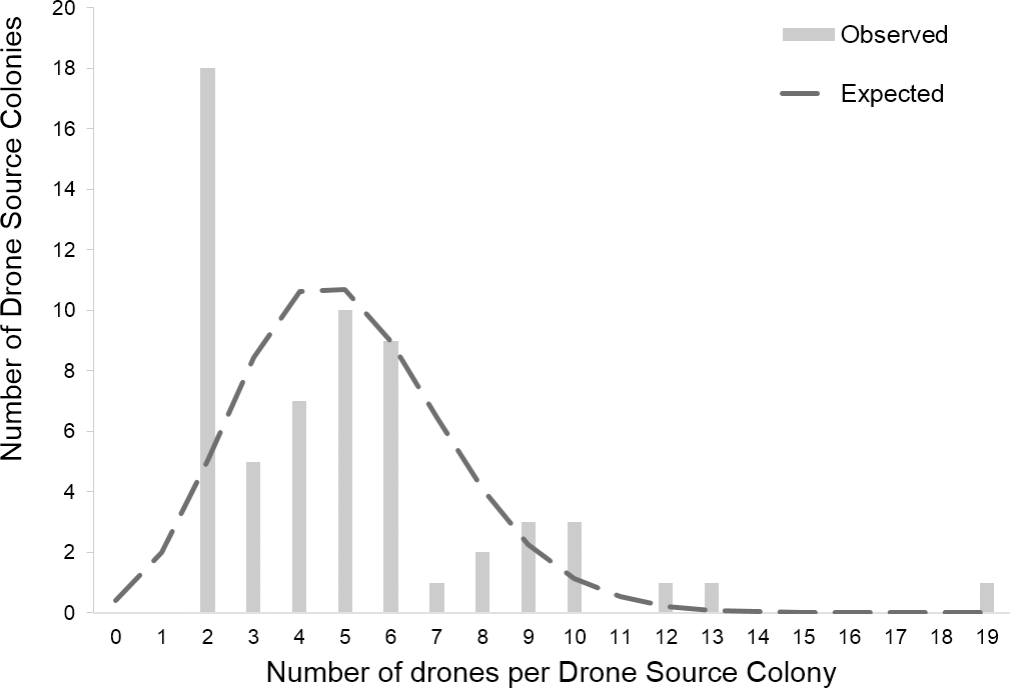
Estimation of the non-sampling error of the number of Drone Source Colonies (i.e. the number of non-sampled colonies) through a fitted Poisson distribution for honeybee (*Apis mellifera*) mating. Observed frequencies are plotted in bars, expected frequencies (fitted Poisson distribution) are plotted in grey solid line. Here, singletons were paired to estimate the minimum number of Drone Source Colony. The number of non-detected Drone Source Colonies is 0.40.

**Fig 4:**
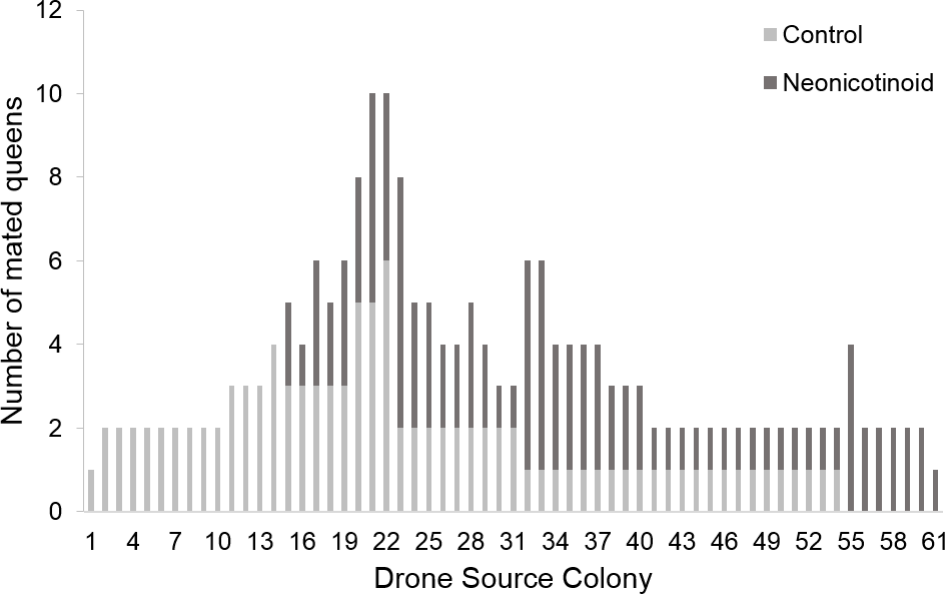
Frequency of control and neonicotinoid honeybee (*Apis mellifera*) queens mated by Drones Source Colony. Singletons that mated with different types of queen (i.e. control or neonicotinoid) were paired. Frequency of queens that mated with drones from each Drone Source Colony is represented in light grey and dark grey for the control and neonicotinoid queens, respectively.

No difference in pollen patty consumption was observed between neonicotinoid (median ± 95% CI = 159.12 ± 113.51–171.78 g) and control (148.94 ± 118.62–168.92 g, menbreg: P = 0.46) colonies. The non-detection error for not discriminating between the genotypes of two siring drones because they share the same genotype by chance was *NDE* < 0.007 (2.18 drones), thus providing confidence in our data set (S1 Table). Sperm Count observed for neonicotinoid queens (4.11x10^6^ ± 1.68) was not significantly different from controls (4.84x10^6^ ± 1.40, meglm: P = 0.15, Table 3). Genotyping estimated that 316 father drones mated with the experimental queens. There was a similar sperm presentation (i.e. post-copulatory sperm mixing) of the various drones since Paternity Skew did not differ between the control (0.34 ± 0.01) and the neonicotinoid queens (0.35 ± 0.03, meglm: P = 0.63, Table 3). The Observed and Corrected Effective Mating were significantly higher for control queens (Observed Effective Mating: 11.72 ± 2.44, median (95% CI); Corrected Effective Mating: 22.11 (18.54, 33.93)) compared to the neonicotinoid ones (Observed Effective Mating: 9.19 ± 1.97, meglm: P = 0.0004, Table 3; median (95% CI); Corrected Effective Mating: 14.01 (11.61, 18.28), mepoisson: P = 0.0023, Fig 1). Furthermore, Observed and Corrected Relatedness were significantly lower for the offspring of control colonies (Observed Relatedness: 0.29 ± 0.01, Corrected Relatedness: 0.27 ± 0.01) than for those of the neonicotinoid colonies (median (95% CI): Observed Relatedness: 0.31 ± 0.01, mepoisson: P = 0.0004, Table 3; Corrected Relatedness: 0.29 ± 0.01, meglm: P = 0.0027, Fig 2). Nine father drones of the 316 identified could not be assigned unambiguously to a Drone Source Colony. The estimated number of Drone Source Colonies ranged from a minimum of 61.40 to a maximum of 64.52, with less than one colony remaining undetected (non-sampling errors = 0.40 and 0.52, respectively, Fig 3). When considering singletons originating from a unique colony, we obtained 18, 11 and 35 colonies that produced drones that mated with queens from controls, neonicotinoids, and both groups, respectively. When pairing singletons that mated with different treatment group queens, we obtained 16, 10, and 35 colonies that produced drones that mated with queens from controls, neonicotinoid, and both groups, respectively. However, when pairing singletons that mated with the same type of queen, we obtained 14, 7, and 40 colonies that produced drones that mated with queens from controls, neonicotinoids, and both groups, respectively (Fig 4). In any estimation, Drone Source Colonies contributed evenly to the mating of the queens (Fisher’s exact test: P > 0.2).

**Table 3:** Effective Mating, Relatedness, Sperm Count and Paternity Skew in the control and neonicotinoid treated honeybee (*Apis mellifera*) queens.

	Colony	Colony source	N	Observed Effective Mating	Observed Relatedness	Corrected Effective Mating	Corrected Relatedness	Sperm Count (x10^6^)	Paternity skew
**Control**	1	1C	24	11.62	0.29	19.71	0.27	4.78	0.35
	2	1C	24	8.73	0.31	13.14	0.29	2.85	0.22
	3	1C	24	11.52	0.29	21.23	0.27	5.78	0.32
	4	1C	24	14.40	0.28	34.50	0.26	5.65	0.33
	5	1C	24	10.67	0.30	18.40	0.28	3.13	0.38
	6	3C	24	13.40	0.29	29.13	0.27	2.32	0.38
	7	3C	20	10.53	0.30	21.11	0.27	6.45	0.36
	8	3C	24	12.00	0.29	23.00	0.27	5.75	0.35
	9	3C	22	6.72	0.32	9.24	0.30	6.50	0.35
	10	5C	24	14.40	0.28	34.50	0.26	5.55	0.33
	11	5C	22	15.13	0.28	46.20	0.26	4.95	0.32
	12	5C	22	11.52	0.29	23.10	0.27	4.32	0.34
			**Mean**	11.72	0.29	24.44	0.27	4.84	0.34
			**± SD**	± 2.44	± 0.01	± 10.18	± 0.01	± 1.40	± 0.01
**Neonicotinoid**	1	2P	24	8.73	0.31	13.14	0.29	2.95	0.32
	2	2P	24	9.93	0.30	16.23	0.28	4.77	0.36
	3	2P	24	10.67	0.30	18.40	0.28	4.44	0.31
	4	4P	24	13.09	0.29	27.60	0.27	0.87	0.22
	5	4P	22	8.22	0.31	12.57	0.29	5.30	0.37
	6	4P	23	9.28	0.30	14.88	0.28	2.37	0.27
	7	4P	24	10.29	0.30	17.25	0.28	3.75	0.34
	8	4P	20	5.26	0.34	6.79	0.32	4.97	0.33
	9	6P	23	10.8	0.30	19.46	0.28	4.07	0.28
	10	6P	24	8.00	0.31	11.50	0.29	7.25	0.38
	11	6P	24	7.58	0.32	10.61	0.30	3.00	0.48
	12	6P	24	8.47	0.31	12.54	0.29	5.65	0.56
			**Mean**	9.19	0.31	15.08	0.29	4.11	0.35
			**± SD**	± 1.97	± 0.01	± 5.32	± 0.01	± 1.68	± 0.03

Observed Effective Mating = number of male mates if all are equally represented in the queen’s offspring; Corrected Effective Mating = Observed Effective Mating corrected for sampling size; Observed Relatedness = intracolonial genetic relatedness; Corrected Relatedness = intracolonial genetic relatedness corrected for sampling size.

## Discussion

Successful mating of the honeybee queen is paramount to colony health and fitness [36]. Our results demonstrate that queens exposed to neonicotinoids during development mated with significantly fewer drones at the same DCAs. Previous studies have reported the negative effects of neonicotinoids on cognition, behaviour, and physiology of honeybees [9,16,73]; however, this is the first observation that neonicotinoids can affect honeybee intracolonial genetic diversity by reducing mating frequency. Since queens only mate during a brief period soon after emergence, the ensuing reduction in genetic diversity of honeybee colonies will continue until the death of the queen or the colony. Therefore, the potential negative effects of neonicotinoids may last many years after initial exposure.

Many possible mechanisms can non-exclusively explain the reduced number of queen matings caused by exposure to neonicotinoids, including behavioural, physiological, or anatomical impairment of queens [48]. Although not all mechanisms guiding the queen’s flight to the DCA are fully understood, it is clear that these require superb cognitive and physiological performance by the queen to locate the DCA and to subsequently return to the colony [25]. It is possible that those orientation skills may be susceptible to known neurotoxic effects of neonicotinoids. Nevertheless, according to our results and Williams et al. [48], queens exposed to neonicotinoids did not exhibit impaired orientation. Indeed, the identified Drone Source Colonies suggest that control and neonicotinoid queens mated in the same DCAs. It could be that potential differences in queen pheromone bouquets [23] may have reduced the attraction of neonicotinoid queens to drones once at these mating areas. Since we did not observe significant differences in sperm counts, muscles responsible for moving drone spermatozoa from the oviducts to the spermathecal [31] did not appear to be impaired. More research is needed to understand this phenomenon, as well as the potential effects of neonicotinoids on aspects of the nervous system responsible for sperm movement and storage.

Our analyses represent a snap-shot of the intracolonial worker patriline distribution in time. Although the frequency of various subfamilies may vary over time [74], the total number of sub-families does not because the queen does not mate once she has started to oviposit [23]. In addition, further studies focusing on each pesticide separately are required to assess single exposure scenarios.

Intracolony genetic diversity generated by polyandry is an important fitness parameter that contributes to enhanced colony survival and disease resistance [40]. Although we did not test colony level traits, any reduction in the number of effective matings results in a reduced colony-level genetic diversity. The latter has been shown to affect colony productivity and survival, and therefore may represent a possible cryptic threat to honeybee colony health [41] in addition to the suite of pests and pathogens that may also affect honeybees [75,76].

## Conclusions

Our data suggest that combined exposure to the neonicotinoids thiamethoxam and clothianidin can have a negative long term effect on colony health by reducing intracolonial genetic diversity resulting from few matings. The data highlight an important sublethal effect of neonicotinoids for eusocial species relying on one or few primary reproductives [25].

## Supporting information

S1 Table Raw data of the worker offspring genotyping for each mated honeybee queens (*Apis mellifera*). Tweenty to 24 worker offspring (individuals) per queen were genotyped using five closely linked microsatellite loci (HB007, HB005, HB004, SV240 and HB15). Alleles were scored as fragment lengths in base pairs. Colony source refers to the colony from which queens were reared. Treatments are noted “P” when the queens were exposed to neonicotinoids (thiamethoxam and clothianidin) during developmental stage, or “C” for controls.
(DOCX)
