# Supplementary material for: Neonicotinoid pesticides can reduce honeybee colony genetic diversity
Source: PLoS One. 2017 Oct 23;12(10):e0186109. doi: 10.1371/journal.pone.0186109 (PMC5653293; doi:10.1371/journal.pone.0186109)
Supplement: S1 Table — Tweenty to 24 worker offspring (individuals) per queen were genotyped using five closely linked microsatellite loci (HB007, HB005, HB004, SV240 and HB15). Alleles were scored as fragment lengths in base pairs. Colony source refers to the colony from which queens were reared. Treatments are noted “P” when the queens were exposed to neonicotinoids (thiamethoxam and clothianidin) during developmental stage, or “C” for controls. (DOCX) [file pone.0186109.s001.docx]

| Queen | Colony source | Treatment | Individual | HB007 | HB007 | HB005 | HB005 | HB004 | HB004 | SV240 | SV240 | HB15 | HB15 |
| --- | --- | --- | --- | --- | --- | --- | --- | --- | --- | --- | --- | --- | --- |
| 6 | 4P | P | 1 | 204 | 216 | 0 | 0 | 180 | 180 | 266 | 278 | 103 | 117 |
| 6 | 4P | P | 2 | 186 | 188 | 223 | 227 | 180 | 184 | 266 | 266 | 103 | 115 |
| 6 | 4P | P | 3 | 186 | 188 | 223 | 227 | 182 | 184 | 266 | 266 | 103 | 115 |
| 6 | 4P | P | 4 | 184 | 204 | 219 | 227 | 180 | 182 | 266 | 266 | 103 | 115 |
| 6 | 4P | P | 5 | 186 | 188 | 223 | 227 | 182 | 184 | 266 | 266 | 103 | 115 |
| 6 | 4P | P | 6 | 154 | 204 | 217 | 227 | 180 | 184 | 266 | 278 | 103 | 115 |
| 6 | 4P | P | 7 | 194 | 204 | 223 | 227 | 182 | 184 | 266 | 266 | 103 | 115 |
| 6 | 4P | P | 8 | 186 | 186 | 185 | 227 | 182 | 182 | 266 | 266 | 103 | 115 |
| 6 | 4P | P | 9 | 186 | 194 | 223 | 227 | 182 | 184 | 266 | 266 | 103 | 115 |
| 6 | 4P | P | 10 | 160 | 204 | 193 | 227 | 0 | 0 | 266 | 266 | 103 | 115 |
| 6 | 4P | P | 11 | 186 | 188 | 223 | 227 | 182 | 184 | 266 | 266 | 103 | 115 |
| 6 | 4P | P | 12 | 0 | 0 | 193 | 227 | 0 | 0 | 0 | 0 | 103 | 115 |
| 6 | 4P | P | 13 | 0 | 0 | 0 | 0 | 0 | 0 | 0 | 0 | 103 | 117 |
| 6 | 4P | P | 14 | 160 | 204 | 0 | 0 | 178 | 180 | 266 | 266 | 103 | 115 |
| 6 | 4P | P | 15 | 186 | 194 | 223 | 227 | 182 | 184 | 266 | 266 | 103 | 115 |
| 6 | 4P | P | 16 | 154 | 186 | 217 | 227 | 182 | 184 | 266 | 278 | 103 | 115 |
| 6 | 4P | P | 17 | 186 | 192 | 227 | 235 | 180 | 182 | 266 | 266 | 103 | 115 |
| 6 | 4P | P | 18 | 186 | 204 | 227 | 235 | 180 | 182 | 266 | 266 | 103 | 115 |
| 6 | 4P | P | 19 | 166 | 204 | 185 | 227 | 180 | 182 | 266 | 266 | 103 | 115 |
| 6 | 4P | P | 20 | 184 | 204 | 219 | 227 | 180 | 180 | 266 | 266 | 103 | 115 |
| 6 | 4P | P | 21 | 186 | 204 | 185 | 227 | 182 | 182 | 266 | 266 | 103 | 115 |
| 6 | 4P | P | 22 | 186 | 204 | 227 | 227 | 180 | 182 | 266 | 266 | 103 | 115 |
| 6 | 4P | P | 23 | 186 | 204 | 227 | 227 | 180 | 182 | 266 | 266 | 103 | 115 |
| 6 | 4P | P | 24 | 0 | 0 | 0 | 0 | 0 | 0 | 0 | 0 | 0 | 0 |
| 7 | 4P | P | 1 | 190 | 204 | 0 | 0 | 0 | 0 | 266 | 266 | 103 | 115 |
| 7 | 4P | P | 2 | 186 | 186 | 0 | 0 | 182 | 182 | 266 | 266 | 103 | 115 |
| 7 | 4P | P | 3 | 186 | 216 | 207 | 207 | 176 | 182 | 266 | 266 | 103 | 117 |
| 7 | 4P | P | 4 | 154 | 204 | 0 | 0 | 180 | 182 | 266 | 266 | 0 | 0 |
| 7 | 4P | P | 5 | 186 | 204 | 205 | 207 | 0 | 0 | 266 | 290 | 103 | 115 |
| 7 | 4P | P | 6 | 186 | 216 | 207 | 207 | 176 | 182 | 266 | 266 | 103 | 117 |
| 7 | 4P | P | 7 | 186 | 204 | 205 | 227 | 182 | 184 | 266 | 266 | 103 | 111 |
| 7 | 4P | P | 8 | 154 | 186 | 0 | 0 | 182 | 182 | 266 | 266 | 103 | 115 |
| 7 | 4P | P | 9 | 186 | 190 | 0 | 0 | 0 | 0 | 266 | 266 | 103 | 115 |
| 7 | 4P | P | 10 | 166 | 204 | 0 | 0 | 180 | 182 | 266 | 266 | 103 | 115 |
| 7 | 4P | P | 11 | 190 | 204 | 0 | 0 | 180 | 180 | 266 | 278 | 103 | 115 |
| 7 | 4P | P | 12 | 154 | 186 | 0 | 0 | 182 | 182 | 266 | 266 | 103 | 103 |
| 7 | 4P | P | 13 | 204 | 216 | 0 | 0 | 176 | 180 | 266 | 266 | 0 | 0 |
| 7 | 4P | P | 14 | 188 | 204 | 213 | 227 | 182 | 182 | 266 | 270 | 0 | 0 |
| 7 | 4P | P | 15 | 190 | 204 | 0 | 0 | 180 | 180 | 266 | 278 | 103 | 115 |
| 7 | 4P | P | 16 | 204 | 204 | 205 | 227 | 180 | 184 | 266 | 266 | 0 | 0 |
| 7 | 4P | P | 17 | 186 | 194 | 207 | 207 | 176 | 182 | 266 | 266 | 103 | 115 |
| 7 | 4P | P | 18 | 188 | 204 | 213 | 227 | 180 | 182 | 266 | 270 | 103 | 115 |
| 7 | 4P | P | 19 | 186 | 216 | 207 | 207 | 176 | 182 | 266 | 266 | 103 | 117 |
| 7 | 4P | P | 20 | 174 | 204 | 0 | 0 | 176 | 180 | 266 | 266 | 0 | 0 |
| 7 | 4P | P | 21 | 194 | 204 | 207 | 207 | 176 | 182 | 266 | 266 | 103 | 115 |
| 7 | 4P | P | 22 | 194 | 204 | 207 | 227 | 176 | 182 | 266 | 266 | 103 | 115 |
| 7 | 4P | P | 23 | 186 | 190 | 0 | 0 | 180 | 182 | 266 | 278 | 103 | 115 |
| 7 | 4P | P | 24 | 186 | 186 | 0 | 0 | 182 | 182 | 0 | 0 | 103 | 103 |
| 8 | 4P | P | 1 | 186 | 194 | 0 | 0 | 0 | 0 | 266 | 266 | 103 | 103 |
| 8 | 4P | P | 2 | 186 | 186 | 0 | 0 | 0 | 0 | 266 | 266 | 103 | 103 |
| 8 | 4P | P | 3 | 154 | 180 | 0 | 0 | 178 | 180 | 266 | 266 | 115 | 115 |
| 8 | 4P | P | 4 | 154 | 180 | 0 | 0 | 178 | 184 | 266 | 266 | 115 | 115 |
| 8 | 4P | P | 5 | 180 | 188 | 0 | 0 | 178 | 182 | 266 | 278 | 103 | 115 |
| 8 | 4P | P | 6 | 0 | 0 | 0 | 0 | 0 | 0 | 0 | 0 | 0 | 0 |
| 8 | 4P | P | 7 | 154 | 186 | 0 | 0 | 182 | 184 | 266 | 266 | 103 | 115 |
| 8 | 4P | P | 8 | 154 | 186 | 0 | 0 | 180 | 182 | 266 | 266 | 115 | 115 |
| 8 | 4P | P | 9 | 186 | 194 | 0 | 0 | 0 | 0 | 266 | 266 | 103 | 115 |
| 8 | 4P | P | 10 | 154 | 180 | 0 | 0 | 178 | 184 | 266 | 266 | 115 | 115 |
| 8 | 4P | P | 11 | 154 | 186 | 0 | 0 | 182 | 184 | 266 | 266 | 103 | 115 |
| 8 | 4P | P | 12 | 0 | 0 | 0 | 0 | 0 | 0 | 0 | 0 | 0 | 0 |
| 8 | 4P | P | 13 | 180 | 194 | 0 | 0 | 178 | 178 | 266 | 266 | 103 | 115 |
| 8 | 4P | P | 14 | 186 | 192 | 0 | 0 | 178 | 182 | 266 | 266 | 103 | 103 |
| 8 | 4P | P | 15 | 186 | 192 | 0 | 0 | 178 | 182 | 266 | 266 | 103 | 103 |
| 8 | 4P | P | 16 | 154 | 186 | 0 | 0 | 180 | 182 | 266 | 266 | 103 | 115 |
| 8 | 4P | P | 17 | 186 | 186 | 0 | 0 | 180 | 182 | 266 | 266 | 103 | 115 |
| 8 | 4P | P | 18 | 154 | 180 | 0 | 0 | 178 | 184 | 266 | 266 | 115 | 115 |
| 8 | 4P | P | 19 | 154 | 186 | 0 | 0 | 180 | 182 | 266 | 266 | 103 | 115 |
| 8 | 4P | P | 20 | 154 | 186 | 0 | 0 | 182 | 184 | 266 | 266 | 103 | 115 |
| 8 | 4P | P | 21 | 0 | 0 | 0 | 0 | 0 | 0 | 0 | 0 | 0 | 0 |
| 8 | 4P | P | 22 | 166 | 186 | 0 | 0 | 182 | 182 | 266 | 276 | 103 | 115 |
| 8 | 4P | P | 23 | 180 | 192 | 0 | 0 | 178 | 182 | 266 | 266 | 103 | 115 |
| 8 | 4P | P | 24 | 0 | 0 | 0 | 0 | 0 | 0 | 0 | 0 | 0 | 0 |
| 9 | 6P | P | 1 | 154 | 190 | 0 | 0 | 180 | 184 | 266 | 266 | 103 | 115 |
| 9 | 6P | P | 2 | 190 | 190 | 205 | 227 | 184 | 184 | 266 | 278 | 103 | 117 |
| 9 | 6P | P | 3 | 186 | 186 | 227 | 227 | 182 | 206 | 266 | 266 | 103 | 103 |
| 9 | 6P | P | 4 | 166 | 186 | 227 | 227 | 180 | 182 | 266 | 276 | 115 | 115 |
| 9 | 6P | P | 5 | 166 | 186 | 205 | 227 | 180 | 182 | 266 | 276 | 115 | 115 |
| 9 | 6P | P | 6 | 166 | 190 | 205 | 219 | 182 | 182 | 266 | 266 | 103 | 115 |
| 9 | 6P | P | 7 | 154 | 186 | 205 | 227 | 182 | 182 | 266 | 276 | 103 | 115 |
| 9 | 6P | P | 8 | 186 | 190 | 225 | 227 | 180 | 182 | 266 | 278 | 103 | 117 |
| 9 | 6P | P | 9 | 186 | 190 | 203 | 227 | 182 | 184 | 266 | 278 | 103 | 103 |
| 9 | 6P | P | 10 | 186 | 190 | 227 | 227 | 184 | 184 | 266 | 278 | 103 | 117 |
| 9 | 6P | P | 11 | 166 | 190 | 227 | 227 | 180 | 184 | 266 | 278 | 115 | 115 |
| 9 | 6P | P | 12 | 190 | 190 | 203 | 205 | 182 | 184 | 266 | 266 | 103 | 117 |
| 9 | 6P | P | 13 | 166 | 186 | 203 | 227 | 182 | 184 | 266 | 278 | 115 | 115 |
| 9 | 6P | P | 14 | 166 | 190 | 203 | 205 | 182 | 184 | 266 | 278 | 115 | 115 |
| 9 | 6P | P | 15 | 186 | 190 | 203 | 227 | 182 | 184 | 266 | 278 | 103 | 103 |
| 9 | 6P | P | 16 | 166 | 190 | 203 | 205 | 182 | 184 | 266 | 278 | 115 | 115 |
| 9 | 6P | P | 17 | 190 | 190 | 0 | 0 | 180 | 184 | 266 | 278 | 103 | 117 |
| 9 | 6P | P | 18 | 190 | 194 | 0 | 0 | 180 | 182 | 266 | 278 | 103 | 117 |
| 9 | 6P | P | 19 | 166 | 186 | 203 | 205 | 182 | 184 | 266 | 278 | 115 | 115 |
| 9 | 6P | P | 20 | 154 | 190 | 205 | 209 | 180 | 184 | 266 | 266 | 103 | 115 |
| 9 | 6P | P | 21 | 154 | 190 | 205 | 209 | 180 | 182 | 266 | 266 | 103 | 115 |
| 9 | 6P | P | 22 | 190 | 194 | 0 | 0 | 180 | 184 | 266 | 266 | 103 | 115 |
| 9 | 6P | P | 23 | 0 | 0 | 0 | 0 | 0 | 0 | 0 | 0 | 0 | 0 |
| 9 | 6P | P | 24 | 186 | 190 | 205 | 227 | 182 | 184 | 266 | 266 | 103 | 103 |
| 10 | 6P | P | 1 | 154 | 194 | 185 | 221 | 0 | 0 | 266 | 266 | 103 | 117 |
| 10 | 6P | P | 2 | 182 | 194 | 185 | 219 | 180 | 182 | 266 | 266 | 103 | 103 |
| 10 | 6P | P | 3 | 162 | 194 | 219 | 221 | 182 | 184 | 266 | 266 | 0 | 0 |
| 10 | 6P | P | 4 | 188 | 194 | 0 | 0 | 178 | 182 | 266 | 278 | 0 | 0 |
| 10 | 6P | P | 5 | 192 | 194 | 219 | 221 | 182 | 182 | 266 | 266 | 103 | 103 |
| 10 | 6P | P | 6 | 154 | 166 | 185 | 219 | 180 | 182 | 264 | 266 | 103 | 117 |
| 10 | 6P | P | 7 | 154 | 194 | 0 | 0 | 180 | 182 | 264 | 266 | 103 | 103 |
| 10 | 6P | P | 8 | 188 | 194 | 0 | 0 | 178 | 182 | 266 | 278 | 103 | 117 |
| 10 | 6P | P | 9 | 154 | 182 | 0 | 0 | 180 | 180 | 266 | 266 | 103 | 117 |
| 10 | 6P | P | 10 | 192 | 194 | 0 | 0 | 178 | 180 | 266 | 266 | 103 | 117 |
| 10 | 6P | P | 11 | 154 | 188 | 0 | 0 | 182 | 182 | 266 | 266 | 103 | 117 |
| 10 | 6P | P | 12 | 154 | 154 | 0 | 0 | 180 | 182 | 266 | 266 | 117 | 117 |
| 10 | 6P | P | 13 | 154 | 174 | 0 | 0 | 178 | 180 | 266 | 266 | 103 | 117 |
| 10 | 6P | P | 14 | 154 | 174 | 185 | 219 | 178 | 182 | 266 | 266 | 103 | 103 |
| 10 | 6P | P | 15 | 154 | 188 | 0 | 0 | 182 | 182 | 266 | 266 | 103 | 103 |
| 10 | 6P | P | 16 | 188 | 194 | 0 | 0 | 180 | 182 | 266 | 266 | 103 | 117 |
| 10 | 6P | P | 17 | 154 | 162 | 0 | 0 | 182 | 184 | 266 | 266 | 103 | 117 |
| 10 | 6P | P | 18 | 182 | 194 | 0 | 0 | 180 | 182 | 266 | 266 | 103 | 117 |
| 10 | 6P | P | 19 | 154 | 194 | 185 | 221 | 180 | 180 | 266 | 266 | 103 | 117 |
| 10 | 6P | P | 20 | 194 | 194 | 185 | 219 | 180 | 182 | 264 | 266 | 103 | 103 |
| 10 | 6P | P | 21 | 154 | 194 | 185 | 221 | 180 | 180 | 266 | 266 | 103 | 117 |
| 10 | 6P | P | 22 | 154 | 194 | 219 | 221 | 180 | 180 | 266 | 266 | 103 | 117 |
| 10 | 6P | P | 23 | 188 | 194 | 219 | 221 | 178 | 182 | 266 | 278 | 103 | 117 |
| 10 | 6P | P | 24 | 188 | 194 | 219 | 221 | 0 | 0 | 266 | 266 | 103 | 117 |
| 11 | 6P | P | 1 | 186 | 192 | 0 | 0 | 180 | 182 | 266 | 266 | 103 | 109 |
| 11 | 6P | P | 2 | 186 | 196 | 0 | 0 | 180 | 182 | 266 | 278 | 103 | 111 |
| 11 | 6P | P | 3 | 192 | 194 | 0 | 0 | 176 | 180 | 266 | 266 | 103 | 109 |
| 11 | 6P | P | 4 | 186 | 192 | 0 | 0 | 182 | 182 | 266 | 266 | 103 | 115 |
| 11 | 6P | P | 5 | 186 | 196 | 0 | 0 | 176 | 182 | 266 | 278 | 103 | 111 |
| 11 | 6P | P | 6 | 192 | 194 | 0 | 0 | 182 | 182 | 266 | 266 | 103 | 109 |
| 11 | 6P | P | 7 | 186 | 192 | 0 | 0 | 180 | 182 | 266 | 266 | 103 | 115 |
| 11 | 6P | P | 8 | 186 | 192 | 185 | 207 | 180 | 182 | 266 | 266 | 103 | 115 |
| 11 | 6P | P | 9 | 186 | 194 | 0 | 0 | 182 | 182 | 266 | 266 | 103 | 103 |
| 11 | 6P | P | 10 | 190 | 194 | 185 | 207 | 180 | 182 | 266 | 266 | 103 | 115 |
| 11 | 6P | P | 11 | 186 | 190 | 185 | 207 | 180 | 182 | 266 | 266 | 103 | 115 |
| 11 | 6P | P | 12 | 186 | 192 | 217 | 217 | 182 | 182 | 266 | 266 | 103 | 109 |
| 11 | 6P | P | 13 | 186 | 192 | 0 | 0 | 0 | 0 | 266 | 266 | 103 | 115 |
| 11 | 6P | P | 14 | 192 | 194 | 0 | 0 | 182 | 182 | 266 | 266 | 103 | 115 |
| 11 | 6P | P | 15 | 190 | 194 | 0 | 0 | 182 | 182 | 266 | 266 | 0 | 0 |
| 11 | 6P | P | 16 | 186 | 190 | 0 | 0 | 182 | 182 | 266 | 266 | 103 | 115 |
| 11 | 6P | P | 17 | 192 | 194 | 0 | 0 | 176 | 182 | 266 | 266 | 103 | 109 |
| 11 | 6P | P | 18 | 166 | 194 | 0 | 0 | 180 | 182 | 266 | 278 | 103 | 115 |
| 11 | 6P | P | 19 | 186 | 194 | 185 | 203 | 180 | 182 | 266 | 266 | 103 | 115 |
| 11 | 6P | P | 20 | 188 | 194 | 185 | 203 | 180 | 182 | 266 | 266 | 103 | 115 |
| 11 | 6P | P | 21 | 186 | 186 | 217 | 217 | 180 | 180 | 266 | 266 | 103 | 115 |
| 11 | 6P | P | 22 | 194 | 198 | 217 | 217 | 180 | 182 | 266 | 266 | 103 | 115 |
| 11 | 6P | P | 23 | 186 | 192 | 0 | 0 | 180 | 182 | 266 | 266 | 103 | 115 |
| 11 | 6P | P | 24 | 186 | 186 | 185 | 203 | 180 | 180 | 266 | 266 | 103 | 115 |
| 12 | 6P | P | 1 | 154 | 186 | 0 | 0 | 182 | 182 | 266 | 266 | 103 | 115 |
| 12 | 6P | P | 2 | 190 | 204 | 207 | 207 | 176 | 182 | 266 | 266 | 103 | 115 |
| 12 | 6P | P | 3 | 186 | 192 | 0 | 0 | 178 | 182 | 266 | 266 | 103 | 115 |
| 12 | 6P | P | 4 | 174 | 186 | 205 | 227 | 180 | 182 | 266 | 266 | 103 | 115 |
| 12 | 6P | P | 5 | 186 | 200 | 205 | 219 | 180 | 192 | 264 | 266 | 103 | 115 |
| 12 | 6P | P | 6 | 186 | 192 | 205 | 205 | 180 | 182 | 266 | 266 | 103 | 115 |
| 12 | 6P | P | 7 | 190 | 204 | 0 | 0 | 176 | 192 | 266 | 266 | 103 | 115 |
| 12 | 6P | P | 8 | 190 | 204 | 207 | 207 | 176 | 192 | 266 | 266 | 103 | 115 |
| 12 | 6P | P | 9 | 180 | 186 | 0 | 0 | 176 | 182 | 266 | 266 | 103 | 115 |
| 12 | 6P | P | 10 | 186 | 190 | 0 | 0 | 180 | 192 | 266 | 266 | 0 | 0 |
| 12 | 6P | P | 11 | 174 | 186 | 0 | 0 | 182 | 182 | 266 | 266 | 103 | 103 |
| 12 | 6P | P | 12 | 190 | 196 | 0 | 0 | 182 | 192 | 266 | 278 | 103 | 111 |
| 12 | 6P | P | 13 | 186 | 190 | 0 | 0 | 182 | 182 | 266 | 266 | 103 | 103 |
| 12 | 6P | P | 14 | 190 | 204 | 0 | 0 | 176 | 182 | 266 | 266 | 103 | 115 |
| 12 | 6P | P | 15 | 186 | 216 | 207 | 207 | 178 | 182 | 266 | 266 | 103 | 115 |
| 12 | 6P | P | 16 | 186 | 190 | 205 | 205 | 180 | 182 | 266 | 266 | 103 | 115 |
| 12 | 6P | P | 17 | 186 | 200 | 0 | 0 | 180 | 182 | 266 | 266 | 103 | 115 |
| 12 | 6P | P | 18 | 186 | 192 | 205 | 223 | 180 | 182 | 266 | 266 | 103 | 115 |
| 12 | 6P | P | 19 | 186 | 204 | 0 | 0 | 176 | 182 | 266 | 266 | 103 | 115 |
| 12 | 6P | P | 20 | 186 | 204 | 207 | 207 | 176 | 182 | 266 | 266 | 103 | 115 |
| 12 | 6P | P | 21 | 180 | 186 | 207 | 207 | 182 | 182 | 266 | 266 | 103 | 115 |
| 12 | 6P | P | 22 | 186 | 190 | 205 | 205 | 180 | 182 | 266 | 266 | 103 | 115 |
| 12 | 6P | P | 23 | 186 | 190 | 0 | 0 | 180 | 182 | 266 | 266 | 103 | 115 |
| 12 | 6P | P | 24 | 0 | 0 | 0 | 0 | 182 | 182 | 266 | 278 | 103 | 103 |
| 1 | 1C | C | 1 | 190 | 194 | 0 | 0 | 180 | 182 | 266 | 266 | 0 | 0 |
| 1 | 1C | C | 2 | 174 | 196 | 0 | 0 | 178 | 182 | 266 | 278 | 103 | 115 |
| 1 | 1C | C | 3 | 194 | 196 | 0 | 0 | 182 | 182 | 266 | 278 | 0 | 0 |
| 1 | 1C | C | 4 | 174 | 188 | 185 | 191 | 180 | 182 | 266 | 266 | 103 | 115 |
| 1 | 1C | C | 5 | 174 | 190 | 0 | 0 | 0 | 0 | 266 | 266 | 103 | 115 |
| 1 | 1C | C | 6 | 174 | 196 | 185 | 191 | 180 | 182 | 266 | 266 | 0 | 0 |
| 1 | 1C | C | 7 | 174 | 186 | 0 | 0 | 180 | 182 | 266 | 266 | 103 | 115 |
| 1 | 1C | C | 8 | 154 | 174 | 0 | 0 | 180 | 182 | 266 | 278 | 103 | 103 |
| 1 | 1C | C | 9 | 188 | 194 | 209 | 209 | 182 | 182 | 266 | 266 | 103 | 103 |
| 1 | 1C | C | 10 | 194 | 196 | 0 | 0 | 182 | 182 | 266 | 278 | 103 | 103 |
| 1 | 1C | C | 11 | 174 | 196 | 0 | 0 | 178 | 182 | 266 | 278 | 103 | 103 |
| 1 | 1C | C | 12 | 188 | 194 | 197 | 209 | 182 | 182 | 266 | 266 | 103 | 103 |
| 1 | 1C | C | 13 | 154 | 194 | 185 | 185 | 182 | 182 | 266 | 278 | 103 | 115 |
| 1 | 1C | C | 14 | 188 | 194 | 197 | 209 | 182 | 182 | 266 | 266 | 103 | 103 |
| 1 | 1C | C | 15 | 174 | 196 | 0 | 0 | 180 | 182 | 266 | 276 | 103 | 115 |
| 1 | 1C | C | 16 | 188 | 194 | 197 | 209 | 182 | 182 | 266 | 266 | 103 | 103 |
| 1 | 1C | C | 17 | 174 | 196 | 0 | 0 | 182 | 182 | 0 | 0 | 103 | 103 |
| 1 | 1C | C | 18 | 194 | 196 | 209 | 209 | 182 | 182 | 266 | 266 | 103 | 103 |
| 1 | 1C | C | 19 | 194 | 196 | 191 | 209 | 182 | 182 | 266 | 266 | 103 | 103 |
| 1 | 1C | C | 20 | 188 | 194 | 209 | 209 | 182 | 182 | 266 | 266 | 103 | 103 |
| 1 | 1C | C | 21 | 174 | 196 | 0 | 0 | 0 | 0 | 266 | 278 | 103 | 103 |
| 1 | 1C | C | 22 | 174 | 196 | 0 | 0 | 178 | 180 | 266 | 278 | 103 | 103 |
| 1 | 1C | C | 23 | 174 | 196 | 209 | 209 | 178 | 182 | 266 | 278 | 103 | 103 |
| 1 | 1C | C | 24 | 194 | 196 | 0 | 0 | 178 | 182 | 266 | 278 | 103 | 103 |
| 2 | 1C | C | 1 | 174 | 186 | 203 | 227 | 178 | 180 | 266 | 266 | 103 | 103 |
| 2 | 1C | C | 2 | 184 | 184 | 203 | 229 | 178 | 186 | 266 | 266 | 115 | 115 |
| 2 | 1C | C | 3 | 174 | 186 | 201 | 201 | 178 | 180 | 266 | 266 | 103 | 103 |
| 2 | 1C | C | 4 | 184 | 186 | 203 | 227 | 178 | 180 | 266 | 266 | 103 | 115 |
| 2 | 1C | C | 5 | 184 | 194 | 201 | 201 | 178 | 180 | 266 | 266 | 103 | 103 |
| 2 | 1C | C | 6 | 184 | 188 | 201 | 233 | 180 | 180 | 266 | 266 | 103 | 115 |
| 2 | 1C | C | 7 | 184 | 206 | 203 | 231 | 180 | 180 | 266 | 266 | 103 | 115 |
| 2 | 1C | C | 8 | 174 | 186 | 201 | 201 | 180 | 180 | 266 | 266 | 103 | 115 |
| 2 | 1C | C | 9 | 184 | 186 | 201 | 233 | 178 | 180 | 266 | 266 | 103 | 103 |
| 2 | 1C | C | 10 | 174 | 188 | 201 | 233 | 178 | 180 | 266 | 266 | 103 | 103 |
| 2 | 1C | C | 11 | 184 | 186 | 201 | 233 | 178 | 180 | 266 | 266 | 103 | 103 |
| 2 | 1C | C | 12 | 184 | 194 | 203 | 231 | 0 | 0 | 266 | 266 | 103 | 103 |
| 2 | 1C | C | 13 | 184 | 206 | 203 | 231 | 0 | 0 | 266 | 266 | 103 | 115 |
| 2 | 1C | C | 14 | 184 | 206 | 203 | 231 | 180 | 180 | 266 | 266 | 103 | 103 |
| 2 | 1C | C | 15 | 184 | 194 | 203 | 231 | 180 | 180 | 266 | 266 | 103 | 115 |
| 2 | 1C | C | 16 | 184 | 184 | 203 | 229 | 178 | 186 | 266 | 266 | 115 | 115 |
| 2 | 1C | C | 17 | 174 | 206 | 203 | 231 | 180 | 180 | 266 | 266 | 103 | 103 |
| 2 | 1C | C | 18 | 174 | 188 | 0 | 0 | 178 | 180 | 266 | 266 | 103 | 115 |
| 2 | 1C | C | 19 | 174 | 206 | 203 | 235 | 178 | 180 | 266 | 266 | 103 | 115 |
| 2 | 1C | C | 20 | 174 | 194 | 201 | 201 | 180 | 180 | 266 | 266 | 103 | 115 |
| 2 | 1C | C | 21 | 184 | 186 | 201 | 227 | 180 | 180 | 266 | 266 | 103 | 103 |
| 2 | 1C | C | 22 | 166 | 174 | 203 | 207 | 178 | 180 | 266 | 278 | 115 | 115 |
| 2 | 1C | C | 23 | 166 | 174 | 201 | 207 | 178 | 180 | 266 | 278 | 115 | 115 |
| 2 | 1C | C | 24 | 184 | 186 | 203 | 227 | 180 | 180 | 266 | 266 | 103 | 103 |
| 3 | 1C | C | 1 | 186 | 192 | 0 | 0 | 180 | 182 | 266 | 266 | 103 | 115 |
| 3 | 1C | C | 2 | 166 | 184 | 0 | 0 | 0 | 0 | 256 | 266 | 103 | 115 |
| 3 | 1C | C | 3 | 154 | 184 | 0 | 0 | 180 | 188 | 266 | 266 | 103 | 115 |
| 3 | 1C | C | 4 | 184 | 190 | 0 | 0 | 180 | 182 | 266 | 266 | 103 | 115 |
| 3 | 1C | C | 5 | 154 | 192 | 0 | 0 | 180 | 182 | 266 | 266 | 103 | 115 |
| 3 | 1C | C | 6 | 154 | 184 | 0 | 0 | 180 | 182 | 266 | 266 | 103 | 115 |
| 3 | 1C | C | 7 | 174 | 184 | 0 | 0 | 178 | 180 | 266 | 266 | 103 | 115 |
| 3 | 1C | C | 8 | 154 | 184 | 0 | 0 | 180 | 186 | 266 | 270 | 103 | 115 |
| 3 | 1C | C | 9 | 166 | 184 | 0 | 0 | 180 | 180 | 266 | 266 | 103 | 115 |
| 3 | 1C | C | 10 | 184 | 188 | 0 | 0 | 0 | 0 | 266 | 270 | 103 | 115 |
| 3 | 1C | C | 11 | 184 | 186 | 0 | 0 | 180 | 182 | 266 | 266 | 103 | 115 |
| 3 | 1C | C | 12 | 188 | 192 | 0 | 0 | 180 | 180 | 266 | 270 | 0 | 0 |
| 3 | 1C | C | 13 | 184 | 216 | 190 | 190 | 180 | 182 | 266 | 276 | 103 | 103 |
| 3 | 1C | C | 14 | 154 | 192 | 190 | 190 | 180 | 188 | 266 | 266 | 103 | 115 |
| 3 | 1C | C | 15 | 180 | 184 | 185 | 185 | 180 | 184 | 266 | 266 | 103 | 103 |
| 3 | 1C | C | 16 | 166 | 192 | 190 | 207 | 0 | 0 | 266 | 266 | 103 | 115 |
| 3 | 1C | C | 17 | 188 | 192 | 185 | 185 | 180 | 194 | 266 | 270 | 103 | 103 |
| 3 | 1C | C | 18 | 166 | 192 | 190 | 207 | 180 | 180 | 266 | 266 | 103 | 103 |
| 3 | 1C | C | 19 | 184 | 216 | 0 | 0 | 180 | 182 | 266 | 276 | 0 | 0 |
| 3 | 1C | C | 20 | 184 | 190 | 0 | 0 | 180 | 182 | 266 | 266 | 103 | 115 |
| 3 | 1C | C | 21 | 190 | 192 | 0 | 0 | 180 | 182 | 266 | 266 | 103 | 115 |
| 3 | 1C | C | 22 | 174 | 192 | 0 | 0 | 178 | 180 | 266 | 270 | 103 | 111 |
| 3 | 1C | C | 23 | 184 | 216 | 0 | 0 | 180 | 182 | 266 | 276 | 103 | 103 |
| 3 | 1C | C | 24 | 154 | 184 | 0 | 0 | 180 | 188 | 266 | 266 | 0 | 0 |
| 4 | 1C | C | 1 | 0 | 0 | 0 | 0 | 0 | 0 | 0 | 0 | 109 | 115 |
| 4 | 1C | C | 2 | 166 | 190 | 0 | 0 | 0 | 0 | 266 | 276 | 109 | 115 |
| 4 | 1C | C | 3 | 166 | 190 | 0 | 0 | 180 | 184 | 264 | 266 | 103 | 115 |
| 4 | 1C | C | 4 | 190 | 194 | 0 | 0 | 182 | 184 | 266 | 266 | 103 | 115 |
| 4 | 1C | C | 5 | 190 | 190 | 0 | 0 | 180 | 184 | 266 | 266 | 111 | 115 |
| 4 | 1C | C | 6 | 190 | 196 | 0 | 0 | 178 | 184 | 266 | 278 | 103 | 115 |
| 4 | 1C | C | 7 | 174 | 174 | 0 | 0 | 180 | 186 | 256 | 266 | 103 | 115 |
| 4 | 1C | C | 8 | 190 | 190 | 0 | 0 | 182 | 184 | 266 | 266 | 103 | 115 |
| 4 | 1C | C | 9 | 174 | 174 | 0 | 0 | 182 | 180 | 266 | 266 | 103 | 115 |
| 4 | 1C | C | 10 | 188 | 190 | 0 | 0 | 180 | 184 | 266 | 266 | 103 | 115 |
| 4 | 1C | C | 11 | 174 | 194 | 0 | 0 | 180 | 180 | 266 | 266 | 103 | 103 |
| 4 | 1C | C | 12 | 174 | 174 | 0 | 0 | 180 | 182 | 266 | 278 | 103 | 103 |
| 4 | 1C | C | 13 | 186 | 190 | 0 | 0 | 180 | 182 | 266 | 266 | 103 | 115 |
| 4 | 1C | C | 14 | 174 | 190 | 0 | 0 | 180 | 184 | 266 | 266 | 103 | 115 |
| 4 | 1C | C | 15 | 174 | 174 | 0 | 0 | 180 | 186 | 258 | 266 | 103 | 115 |
| 4 | 1C | C | 16 | 190 | 190 | 0 | 0 | 184 | 186 | 258 | 266 | 111 | 115 |
| 4 | 1C | C | 17 | 188 | 190 | 0 | 0 | 180 | 184 | 266 | 266 | 103 | 115 |
| 4 | 1C | C | 18 | 174 | 194 | 0 | 0 | 180 | 184 | 266 | 266 | 103 | 103 |
| 4 | 1C | C | 19 | 174 | 196 | 0 | 0 | 178 | 180 | 266 | 278 | 103 | 103 |
| 4 | 1C | C | 20 | 166 | 174 | 0 | 0 | 180 | 182 | 256 | 266 | 103 | 115 |
| 4 | 1C | C | 21 | 174 | 192 | 0 | 0 | 180 | 182 | 266 | 266 | 103 | 115 |
| 4 | 1C | C | 22 | 186 | 190 | 0 | 0 | 182 | 184 | 266 | 266 | 103 | 115 |
| 4 | 1C | C | 23 | 190 | 196 | 0 | 0 | 178 | 184 | 266 | 278 | 103 | 115 |
| 4 | 1C | C | 24 | 174 | 174 | 0 | 0 | 180 | 182 | 266 | 278 | 103 | 115 |
| 5 | 1C | C | 1 | 186 | 192 | 201 | 227 | 178 | 182 | 266 | 266 | 103 | 115 |
| 5 | 1C | C | 2 | 192 | 192 | 201 | 241 | 178 | 180 | 266 | 266 | 103 | 115 |
| 5 | 1C | C | 3 | 166 | 184 | 207 | 213 | 180 | 182 | 266 | 266 | 103 | 115 |
| 5 | 1C | C | 4 | 184 | 192 | 213 | 237 | 180 | 180 | 266 | 266 | 103 | 115 |
| 5 | 1C | C | 5 | 192 | 212 | 213 | 231 | 180 | 182 | 266 | 278 | 103 | 117 |
| 5 | 1C | C | 6 | 192 | 192 | 213 | 237 | 180 | 180 | 266 | 266 | 103 | 115 |
| 5 | 1C | C | 7 | 184 | 192 | 213 | 231 | 180 | 182 | 266 | 278 | 103 | 115 |
| 5 | 1C | C | 8 | 192 | 212 | 201 | 231 | 178 | 182 | 266 | 278 | 103 | 117 |
| 5 | 1C | C | 9 | 166 | 184 | 0 | 0 | 180 | 182 | 266 | 266 | 0 | 0 |
| 5 | 1C | C | 10 | 192 | 192 | 201 | 205 | 178 | 182 | 266 | 266 | 103 | 109 |
| 5 | 1C | C | 11 | 188 | 192 | 201 | 223 | 178 | 184 | 266 | 266 | 0 | 0 |
| 5 | 1C | C | 12 | 184 | 188 | 201 | 209 | 178 | 182 | 266 | 266 | 103 | 115 |
| 5 | 1C | C | 13 | 184 | 192 | 213 | 213 | 178 | 180 | 266 | 266 | 103 | 129 |
| 5 | 1C | C | 14 | 188 | 192 | 201 | 223 | 178 | 184 | 266 | 266 | 103 | 115 |
| 5 | 1C | C | 15 | 184 | 188 | 209 | 213 | 180 | 182 | 266 | 266 | 103 | 115 |
| 5 | 1C | C | 16 | 184 | 212 | 213 | 231 | 180 | 182 | 266 | 278 | 103 | 117 |
| 5 | 1C | C | 17 | 184 | 186 | 201 | 227 | 178 | 182 | 266 | 266 | 103 | 115 |
| 5 | 1C | C | 18 | 192 | 192 | 201 | 221 | 178 | 180 | 266 | 266 | 103 | 115 |
| 5 | 1C | C | 19 | 188 | 192 | 201 | 223 | 178 | 184 | 266 | 266 | 103 | 115 |
| 5 | 1C | C | 20 | 184 | 192 | 201 | 201 | 178 | 180 | 266 | 266 | 103 | 115 |
| 5 | 1C | C | 21 | 184 | 212 | 201 | 231 | 178 | 182 | 266 | 278 | 103 | 117 |
| 5 | 1C | C | 22 | 188 | 192 | 201 | 209 | 178 | 182 | 266 | 266 | 103 | 115 |
| 5 | 1C | C | 23 | 184 | 192 | 213 | 237 | 180 | 180 | 266 | 266 | 103 | 129 |
| 5 | 1C | C | 24 | 192 | 192 | 201 | 239 | 178 | 180 | 266 | 266 | 103 | 129 |
| 6 | 3C | C | 1 | 182 | 190 | 185 | 185 | 180 | 182 | 266 | 266 | 103 | 103 |
| 6 | 3C | C | 2 | 182 | 194 | 185 | 207 | 180 | 182 | 266 | 266 | 103 | 103 |
| 6 | 3C | C | 3 | 182 | 188 | 0 | 0 | 182 | 182 | 266 | 266 | 103 | 103 |
| 6 | 3C | C | 4 | 0 | 0 | 185 | 185 | 182 | 182 | 266 | 278 | 103 | 116 |
| 6 | 3C | C | 5 | 166 | 198 | 185 | 207 | 180 | 182 | 266 | 278 | 103 | 103 |
| 6 | 3C | C | 6 | 154 | 198 | 185 | 207 | 180 | 182 | 266 | 266 | 103 | 103 |
| 6 | 3C | C | 7 | 194 | 198 | 185 | 213 | 180 | 182 | 266 | 278 | 103 | 103 |
| 6 | 3C | C | 8 | 188 | 198 | 185 | 185 | 182 | 182 | 266 | 266 | 103 | 103 |
| 6 | 3C | C | 9 | 190 | 198 | 185 | 207 | 180 | 182 | 266 | 266 | 103 | 103 |
| 6 | 3C | C | 10 | 190 | 198 | 185 | 207 | 180 | 182 | 266 | 266 | 103 | 103 |
| 6 | 3C | C | 11 | 166 | 198 | 185 | 207 | 182 | 182 | 266 | 266 | 103 | 103 |
| 6 | 3C | C | 12 | 182 | 194 | 185 | 185 | 180 | 182 | 266 | 266 | 103 | 103 |
| 6 | 3C | C | 13 | 182 | 198 | 185 | 207 | 182 | 182 | 266 | 266 | 103 | 103 |
| 6 | 3C | C | 14 | 182 | 194 | 185 | 207 | 180 | 182 | 266 | 266 | 103 | 103 |
| 6 | 3C | C | 15 | 182 | 198 | 185 | 207 | 182 | 182 | 266 | 266 | 103 | 103 |
| 6 | 3C | C | 16 | 182 | 194 | 185 | 207 | 180 | 182 | 266 | 266 | 103 | 103 |
| 6 | 3C | C | 17 | 182 | 198 | 185 | 207 | 0 | 0 | 0 | 0 | 103 | 116 |
| 6 | 3C | C | 18 | 166 | 198 | 185 | 207 | 182 | 182 | 266 | 278 | 103 | 103 |
| 6 | 3C | C | 19 | 198 | 198 | 185 | 207 | 182 | 206 | 266 | 266 | 103 | 103 |
| 6 | 3C | C | 20 | 182 | 198 | 185 | 185 | 182 | 218 | 266 | 266 | 103 | 103 |
| 6 | 3C | C | 21 | 182 | 190 | 0 | 0 | 180 | 182 | 266 | 266 | 103 | 103 |
| 6 | 3C | C | 22 | 182 | 190 | 185 | 207 | 180 | 182 | 266 | 266 | 103 | 103 |
| 6 | 3C | C | 23 | 166 | 198 | 185 | 185 | 0 | 0 | 266 | 278 | 103 | 103 |
| 6 | 3C | C | 24 | 154 | 198 | 185 | 185 | 182 | 182 | 266 | 278 | 103 | 103 |
| 7 | 3C | C | 1 | 0 | 0 | 0 | 0 | 0 | 0 | 0 | 0 | 0 | 0 |
| 7 | 3C | C | 2 | 0 | 0 | 0 | 0 | 0 | 0 | 0 | 0 | 0 | 0 |
| 7 | 3C | C | 3 | 0 | 0 | 0 | 0 | 0 | 0 | 0 | 0 | 0 | 0 |
| 7 | 3C | C | 4 | 0 | 0 | 0 | 0 | 0 | 0 | 0 | 0 | 0 | 0 |
| 7 | 3C | C | 5 | 188 | 198 | 203 | 203 | 180 | 182 | 266 | 266 | 103 | 103 |
| 7 | 3C | C | 6 | 198 | 198 | 0 | 0 | 180 | 182 | 266 | 278 | 103 | 129 |
| 7 | 3C | C | 7 | 176 | 188 | 203 | 203 | 176 | 180 | 266 | 266 | 103 | 115 |
| 7 | 3C | C | 8 | 188 | 198 | 203 | 203 | 182 | 182 | 266 | 266 | 103 | 115 |
| 7 | 3C | C | 9 | 176 | 176 | 0 | 0 | 176 | 198 | 266 | 266 | 0 | 0 |
| 7 | 3C | C | 10 | 196 | 198 | 203 | 207 | 182 | 182 | 266 | 266 | 103 | 115 |
| 7 | 3C | C | 11 | 188 | 198 | 0 | 0 | 176 | 180 | 266 | 266 | 103 | 103 |
| 7 | 3C | C | 12 | 176 | 182 | 0 | 0 | 176 | 184 | 266 | 266 | 103 | 111 |
| 7 | 3C | C | 13 | 196 | 198 | 203 | 207 | 182 | 182 | 266 | 278 | 103 | 115 |
| 7 | 3C | C | 14 | 188 | 198 | 203 | 203 | 176 | 180 | 266 | 266 | 103 | 103 |
| 7 | 3C | C | 15 | 176 | 188 | 203 | 203 | 176 | 182 | 266 | 266 | 103 | 115 |
| 7 | 3C | C | 16 | 188 | 198 | 203 | 203 | 180 | 182 | 266 | 266 | 103 | 115 |
| 7 | 3C | C | 17 | 176 | 190 | 0 | 0 | 180 | 182 | 266 | 278 | 103 | 115 |
| 7 | 3C | C | 18 | 196 | 198 | 203 | 207 | 182 | 182 | 266 | 278 | 103 | 115 |
| 7 | 3C | C | 19 | 176 | 188 | 203 | 203 | 176 | 180 | 266 | 266 | 103 | 115 |
| 7 | 3C | C | 20 | 198 | 200 | 203 | 207 | 176 | 180 | 266 | 266 | 103 | 115 |
| 7 | 3C | C | 21 | 176 | 198 | 203 | 203 | 176 | 182 | 266 | 278 | 103 | 115 |
| 7 | 3C | C | 22 | 168 | 198 | 203 | 207 | 182 | 182 | 266 | 266 | 103 | 115 |
| 7 | 3C | C | 23 | 182 | 198 | 203 | 207 | 182 | 184 | 266 | 266 | 103 | 111 |
| 7 | 3C | C | 24 | 190 | 198 | 203 | 207 | 180 | 182 | 266 | 278 | 103 | 103 |
| 8 | 3C | C | 1 | 186 | 192 | 205 | 205 | 182 | 182 | 266 | 266 | 103 | 103 |
| 8 | 3C | C | 2 | 186 | 190 | 185 | 219 | 180 | 180 | 266 | 266 | 103 | 115 |
| 8 | 3C | C | 3 | 186 | 190 | 190 | 205 | 180 | 180 | 266 | 266 | 103 | 115 |
| 8 | 3C | C | 4 | 186 | 190 | 0 | 0 | 180 | 180 | 266 | 266 | 103 | 103 |
| 8 | 3C | C | 5 | 186 | 186 | 0 | 0 | 180 | 180 | 266 | 278 | 103 | 103 |
| 8 | 3C | C | 6 | 166 | 186 | 185 | 185 | 180 | 180 | 258 | 266 | 103 | 115 |
| 8 | 3C | C | 7 | 190 | 192 | 205 | 205 | 182 | 182 | 266 | 266 | 103 | 109 |
| 8 | 3C | C | 8 | 186 | 186 | 0 | 0 | 180 | 180 | 266 | 278 | 103 | 103 |
| 8 | 3C | C | 9 | 166 | 186 | 185 | 205 | 180 | 184 | 266 | 278 | 115 | 115 |
| 8 | 3C | C | 10 | 154 | 186 | 185 | 185 | 182 | 182 | 266 | 278 | 115 | 115 |
| 8 | 3C | C | 11 | 186 | 192 | 185 | 205 | 182 | 182 | 266 | 266 | 109 | 115 |
| 8 | 3C | C | 12 | 154 | 190 | 185 | 185 | 182 | 182 | 266 | 266 | 103 | 115 |
| 8 | 3C | C | 13 | 166 | 186 | 185 | 185 | 180 | 180 | 258 | 266 | 115 | 115 |
| 8 | 3C | C | 14 | 166 | 186 | 185 | 185 | 0 | 0 | 276 | 278 | 115 | 115 |
| 8 | 3C | C | 15 | 166 | 186 | 185 | 185 | 180 | 180 | 266 | 278 | 115 | 115 |
| 8 | 3C | C | 16 | 186 | 186 | 205 | 205 | 180 | 180 | 266 | 266 | 103 | 103 |
| 8 | 3C | C | 17 | 190 | 192 | 205 | 205 | 182 | 182 | 266 | 266 | 103 | 109 |
| 8 | 3C | C | 18 | 186 | 186 | 205 | 219 | 180 | 180 | 266 | 266 | 103 | 103 |
| 8 | 3C | C | 19 | 190 | 192 | 205 | 228 | 182 | 182 | 266 | 266 | 103 | 103 |
| 8 | 3C | C | 20 | 0 | 0 | 205 | 205 | 180 | 180 | 266 | 266 | 103 | 109 |
| 8 | 3C | C | 21 | 186 | 186 | 185 | 205 | 182 | 182 | 266 | 278 | 103 | 103 |
| 8 | 3C | C | 22 | 190 | 192 | 205 | 228 | 180 | 180 | 266 | 266 | 103 | 109 |
| 8 | 3C | C | 23 | 186 | 190 | 185 | 203 | 0 | 0 | 266 | 278 | 103 | 103 |
| 8 | 3C | C | 24 | 166 | 190 | 185 | 185 | 180 | 180 | 258 | 278 | 103 | 115 |
| 9 | 3C | C | 1 | 188 | 198 | 0 | 0 | 182 | 182 | 266 | 266 | 103 | 103 |
| 9 | 3C | C | 2 | 182 | 188 | 203 | 203 | 182 | 182 | 266 | 266 | 103 | 103 |
| 9 | 3C | C | 3 | 182 | 196 | 0 | 0 | 178 | 182 | 266 | 266 | 103 | 111 |
| 9 | 3C | C | 4 | 188 | 198 | 227 | 231 | 178 | 182 | 266 | 266 | 103 | 115 |
| 9 | 3C | C | 5 | 198 | 206 | 0 | 0 | 182 | 182 | 266 | 266 | 103 | 103 |
| 9 | 3C | C | 6 | 182 | 188 | 203 | 203 | 182 | 182 | 266 | 266 | 103 | 103 |
| 9 | 3C | C | 7 | 198 | 206 | 227 | 231 | 178 | 182 | 266 | 266 | 103 | 115 |
| 9 | 3C | C | 8 | 166 | 182 | 185 | 203 | 182 | 182 | 266 | 278 | 103 | 115 |
| 9 | 3C | C | 9 | 182 | 188 | 203 | 227 | 182 | 182 | 266 | 266 | 103 | 103 |
| 9 | 3C | C | 10 | 196 | 198 | 203 | 231 | 178 | 182 | 266 | 266 | 111 | 115 |
| 9 | 3C | C | 11 | 166 | 182 | 185 | 227 | 182 | 182 | 266 | 278 | 115 | 115 |
| 9 | 3C | C | 12 | 0 | 0 | 0 | 0 | 0 | 0 | 0 | 0 | 0 | 0 |
| 9 | 3C | C | 13 | 188 | 198 | 0 | 0 | 182 | 182 | 266 | 266 | 0 | 0 |
| 9 | 3C | C | 14 | 166 | 198 | 0 | 0 | 178 | 182 | 266 | 278 | 115 | 115 |
| 9 | 3C | C | 15 | 182 | 188 | 203 | 227 | 182 | 182 | 266 | 266 | 103 | 115 |
| 9 | 3C | C | 16 | 196 | 198 | 227 | 227 | 182 | 182 | 266 | 266 | 111 | 115 |
| 9 | 3C | C | 17 | 166 | 198 | 0 | 0 | 178 | 182 | 266 | 278 | 103 | 115 |
| 9 | 3C | C | 18 | 198 | 206 | 227 | 227 | 182 | 182 | 266 | 266 | 103 | 103 |
| 9 | 3C | C | 19 | 198 | 206 | 227 | 227 | 178 | 182 | 266 | 266 | 103 | 103 |
| 9 | 3C | C | 20 | 182 | 196 | 227 | 227 | 182 | 182 | 266 | 266 | 111 | 115 |
| 9 | 3C | C | 21 | 182 | 206 | 227 | 227 | 182 | 182 | 266 | 266 | 103 | 103 |
| 9 | 3C | C | 22 | 182 | 206 | 227 | 231 | 178 | 182 | 266 | 266 | 103 | 115 |
| 9 | 3C | C | 23 | 166 | 198 | 185 | 203 | 178 | 182 | 266 | 278 | 103 | 115 |
| 9 | 3C | C | 24 | 0 | 0 | 0 | 0 | 0 | 0 | 0 | 0 | 0 | 0 |
| 10 | 5C | C | 1 | 190 | 216 | 185 | 207 | 176 | 180 | 266 | 266 | 103 | 103 |
| 10 | 5C | C | 2 | 0 | 0 | 185 | 223 | 0 | 0 | 0 | 0 | 103 | 115 |
| 10 | 5C | C | 3 | 190 | 196 | 185 | 227 | 180 | 180 | 266 | 266 | 103 | 103 |
| 10 | 5C | C | 4 | 188 | 190 | 185 | 185 | 178 | 184 | 266 | 278 | 103 | 115 |
| 10 | 5C | C | 5 | 166 | 166 | 185 | 185 | 0 | 0 | 266 | 266 | 103 | 115 |
| 10 | 5C | C | 6 | 190 | 190 | 185 | 185 | 180 | 180 | 266 | 266 | 103 | 115 |
| 10 | 5C | C | 7 | 166 | 190 | 185 | 207 | 180 | 180 | 266 | 266 | 103 | 115 |
| 10 | 5C | C | 8 | 186 | 190 | 185 | 227 | 180 | 180 | 266 | 266 | 103 | 103 |
| 10 | 5C | C | 9 | 166 | 190 | 0 | 0 | 180 | 188 | 266 | 266 | 0 | 0 |
| 10 | 5C | C | 10 | 166 | 174 | 185 | 201 | 178 | 184 | 266 | 266 | 103 | 115 |
| 10 | 5C | C | 11 | 154 | 166 | 185 | 227 | 178 | 180 | 266 | 266 | 115 | 115 |
| 10 | 5C | C | 12 | 166 | 190 | 185 | 185 | 184 | 190 | 266 | 266 | 103 | 115 |
| 10 | 5C | C | 13 | 166 | 190 | 0 | 0 | 180 | 184 | 266 | 266 | 0 | 0 |
| 10 | 5C | C | 14 | 154 | 166 | 185 | 215 | 180 | 184 | 266 | 266 | 115 | 115 |
| 10 | 5C | C | 15 | 188 | 190 | 0 | 0 | 178 | 180 | 266 | 278 | 103 | 103 |
| 10 | 5C | C | 16 | 154 | 166 | 185 | 207 | 176 | 180 | 266 | 266 | 115 | 115 |
| 10 | 5C | C | 17 | 0 | 0 | 185 | 190 | 0 | 0 | 246 | 266 | 115 | 115 |
| 10 | 5C | C | 18 | 162 | 166 | 185 | 223 | 184 | 184 | 266 | 266 | 115 | 115 |
| 10 | 5C | C | 19 | 188 | 190 | 0 | 0 | 180 | 180 | 266 | 270 | 103 | 111 |
| 10 | 5C | C | 20 | 154 | 190 | 185 | 227 | 178 | 180 | 266 | 266 | 103 | 115 |
| 10 | 5C | C | 21 | 166 | 190 | 185 | 231 | 180 | 188 | 266 | 266 | 103 | 115 |
| 10 | 5C | C | 22 | 162 | 190 | 185 | 223 | 180 | 184 | 266 | 266 | 103 | 115 |
| 10 | 5C | C | 23 | 188 | 190 | 0 | 0 | 180 | 180 | 266 | 270 | 103 | 111 |
| 10 | 5C | C | 24 | 166 | 188 | 185 | 227 | 178 | 184 | 266 | 280 | 103 | 115 |
| 11 | 5C | C | 1 | 0 | 0 | 189 | 219 | 0 | 0 | 0 | 0 | 103 | 117 |
| 11 | 5C | C | 2 | 154 | 190 | 219 | 219 | 180 | 192 | 266 | 276 | 117 | 117 |
| 11 | 5C | C | 3 | 182 | 186 | 189 | 219 | 0 | 0 | 266 | 276 | 103 | 111 |
| 11 | 5C | C | 4 | 190 | 190 | 189 | 207 | 180 | 180 | 266 | 266 | 103 | 117 |
| 11 | 5C | C | 5 | 164 | 182 | 201 | 207 | 178 | 180 | 266 | 266 | 103 | 111 |
| 11 | 5C | C | 6 | 0 | 0 | 0 | 0 | 0 | 0 | 0 | 0 | 0 | 0 |
| 11 | 5C | C | 7 | 182 | 190 | 207 | 207 | 180 | 182 | 266 | 266 | 103 | 111 |
| 11 | 5C | C | 8 | 164 | 190 | 201 | 207 | 178 | 180 | 266 | 266 | 103 | 117 |
| 11 | 5C | C | 9 | 190 | 190 | 219 | 233 | 180 | 192 | 266 | 266 | 103 | 117 |
| 11 | 5C | C | 10 | 182 | 194 | 0 | 0 | 180 | 182 | 266 | 266 | 0 | 0 |
| 11 | 5C | C | 11 | 154 | 190 | 0 | 0 | 182 | 192 | 266 | 278 | 103 | 117 |
| 11 | 5C | C | 12 | 0 | 0 | 0 | 0 | 0 | 0 | 0 | 0 | 0 | 0 |
| 11 | 5C | C | 13 | 190 | 190 | 0 | 0 | 182 | 182 | 266 | 266 | 103 | 117 |
| 11 | 5C | C | 14 | 190 | 192 | 201 | 219 | 180 | 192 | 266 | 266 | 103 | 117 |
| 11 | 5C | C | 15 | 164 | 182 | 201 | 207 | 178 | 180 | 266 | 266 | 103 | 111 |
| 11 | 5C | C | 16 | 154 | 190 | 219 | 219 | 182 | 192 | 266 | 278 | 103 | 117 |
| 11 | 5C | C | 17 | 190 | 190 | 207 | 219 | 180 | 192 | 266 | 266 | 103 | 111 |
| 11 | 5C | C | 18 | 154 | 182 | 0 | 0 | 180 | 182 | 266 | 278 | 103 | 111 |
| 11 | 5C | C | 19 | 182 | 190 | 207 | 233 | 180 | 180 | 266 | 266 | 103 | 111 |
| 11 | 5C | C | 20 | 182 | 192 | 207 | 207 | 180 | 182 | 266 | 266 | 103 | 111 |
| 11 | 5C | C | 21 | 154 | 190 | 185 | 207 | 180 | 182 | 266 | 278 | 103 | 117 |
| 11 | 5C | C | 22 | 182 | 194 | 207 | 223 | 180 | 182 | 266 | 266 | 103 | 111 |
| 11 | 5C | C | 23 | 182 | 200 | 207 | 225 | 178 | 180 | 266 | 266 | 103 | 111 |
| 11 | 5C | C | 24 | 0 | 0 | 207 | 219 | 0 | 0 | 266 | 278 | 103 | 111 |
| 12 | 5C | C | 1 | 168 | 182 | 0 | 0 | 180 | 184 | 266 | 266 | 103 | 103 |
| 12 | 5C | C | 2 | 186 | 190 | 207 | 207 | 180 | 180 | 266 | 266 | 103 | 103 |
| 12 | 5C | C | 3 | 166 | 186 | 185 | 207 | 180 | 182 | 266 | 278 | 115 | 115 |
| 12 | 5C | C | 4 | 182 | 216 | 0 | 0 | 176 | 180 | 266 | 266 | 103 | 115 |
| 12 | 5C | C | 5 | 166 | 182 | 207 | 207 | 180 | 180 | 266 | 266 | 103 | 103 |
| 12 | 5C | C | 6 | 182 | 194 | 207 | 223 | 176 | 180 | 266 | 266 | 103 | 103 |
| 12 | 5C | C | 7 | 0 | 0 | 207 | 223 | 182 | 186 | 266 | 266 | 103 | 103 |
| 12 | 5C | C | 8 | 186 | 188 | 207 | 223 | 180 | 180 | 266 | 266 | 103 | 115 |
| 12 | 5C | C | 9 | 168 | 186 | 207 | 223 | 180 | 184 | 266 | 266 | 103 | 103 |
| 12 | 5C | C | 10 | 168 | 182 | 207 | 223 | 180 | 184 | 266 | 266 | 103 | 103 |
| 12 | 5C | C | 11 | 186 | 186 | 207 | 207 | 180 | 182 | 266 | 266 | 0 | 0 |
| 12 | 5C | C | 12 | 0 | 0 | 0 | 0 | 0 | 0 | 0 | 0 | 103 | 103 |
| 12 | 5C | C | 13 | 186 | 188 | 0 | 0 | 180 | 180 | 266 | 266 | 0 | 0 |
| 12 | 5C | C | 14 | 186 | 216 | 207 | 223 | 176 | 180 | 266 | 266 | 103 | 115 |
| 12 | 5C | C | 15 | 166 | 182 | 185 | 207 | 180 | 182 | 266 | 276 | 103 | 103 |
| 12 | 5C | C | 16 | 186 | 194 | 207 | 207 | 176 | 180 | 266 | 266 | 103 | 103 |
| 12 | 5C | C | 17 | 0 | 0 | 0 | 0 | 0 | 0 | 0 | 0 | 0 | 0 |
| 12 | 5C | C | 18 | 166 | 182 | 0 | 0 | 180 | 180 | 266 | 266 | 103 | 103 |
| 12 | 5C | C | 19 | 186 | 196 | 207 | 207 | 180 | 180 | 266 | 266 | 103 | 115 |
| 12 | 5C | C | 20 | 154 | 182 | 185 | 207 | 180 | 180 | 0 | 0 | 103 | 103 |
| 12 | 5C | C | 21 | 186 | 188 | 0 | 0 | 180 | 180 | 266 | 266 | 103 | 103 |
| 12 | 5C | C | 22 | 186 | 190 | 0 | 0 | 180 | 180 | 266 | 266 | 103 | 103 |
| 12 | 5C | C | 23 | 186 | 186 | 207 | 207 | 180 | 182 | 266 | 266 | 103 | 115 |
| 12 | 5C | C | 24 | 166 | 182 | 0 | 0 | 180 | 180 | 266 | 278 | 103 | 117 |
